# Molecular characteristics and potential antigenic epitope analysis of porcine epidemic diarrhea virus in China from 2022 to 2025

**DOI:** 10.3389/fvets.2025.1667063

**Published:** 2025-10-08

**Authors:** Zunbao Wang, Kai Yang, Mingfang Bi, Kaijie Li, Wen Wang, Yan Song, Xiaomei Pan, Tianzeng Li, Xiaobing Mo

**Affiliations:** ^1^State Key Laboratory for Diagnosis and Treatment of Severe Zoonotic Infectious Diseases, Key Laboratory for Zoonosis Research of the Ministry of Education, College of Veterinary Medicine, Institute of Zoonosis, Jilin University, Changchun, China; ^2^Tecon Bio-Pharmaceuticals Co., Ltd., Urumqi, China

**Keywords:** PEDV, S protein, molecular characteristics, mutation analysis, *N*-glycosylation, antigen prediction

## Abstract

Porcine epidemic diarrhea virus (PEDV) causes varying degrees of diarrhea, vomiting, dehydration, and emaciation in pigs, and severe cases can result in the death of suckling piglets. PEDV is one of the significant viruses affecting the global swine industry. In this study, we investigated the prevalence of PEDV infection in 2,346 pig samples collected from 20 provinces in China, with an overall positive rate of 43.3% (1,015/2,346). Phylogenetic analysis of 15 newly sequenced PEDV strains revealed that 1 strain belonged to the G1c (S-INDEL) genotype, 4 strains to G2b, and the remaining 10 strains to G2c, indicating that G2c is currently the dominant subtype. Compared with the classical CV777 strain, the nucleotide and amino acid sequence identities of the *S* gene of these 15 PEDV strains ranged from 92.71 to 94.83% and 92.89 to 94.99%, respectively. Amino acid alignment identified mutations of varying degrees within key neutralizing epitopes, including COE, SS2, SS6, and 2C10. Using the server, a new potential *N*-glycosylation site at position 302 (NKTI) in the S protein of the PEDV/XinJiang/2 strain was identified. Furthermore, linear and discontinuous antigenic epitopes of resolved PEDV proteins were predicted using ElliPro, revealing conserved potential antigenic regions at amino acid 31–54, 90–103, 1,170–1,177, 1,179–1,197, 1,201–1,211, 1,221–1,236, and 1,243–1,254. These findings provide new epidemiological data on PEDV circulating in China and offer valuable insights for the development of future vaccines and molecular diagnostics based on these antigenic epitopes, thereby contributing to improved PED control strategies.

## 1 Introduction

Porcine epidemic diarrhea virus (PEDV) is a single-stranded positive-sense RNA virus belonging to the genus Alphacoronavirus within the family Coronaviridae, which causes porcine epidemic diarrhea (1). Once inside the host, the virus infects the small intestinal mucosa, causing varying degrees of diarrhea, vomiting, dehydration, and emaciation in pigs of all ages, with the highest mortality observed in suckling piglets (2, 3). PEDV was first identified in the 1970s in the United Kingdom and Belgium (4). Subsequently, PEDV was first isolated in 1984 in China (5). The impact of PEDV on pigs in China was mitigated due to the use of inactivated vaccines. However, this relative control was disrupted in 2010, a highly virulent PEDV variant with high mortality emerged (6).

At the molecular level, the full-length PEDV genome is approximately 28 kb in size and consists of a 5′ end, a 5′ cap, a 5′ untranslated region (UTR), seven open reading frames (ORFs), a 3′ untranslated region (UTR), and a polyadenylated (polyA) tail (1). These ORFs encode several structural proteins, including the spike protein (S protein), the membrane protein (M protein), the envelope protein (E protein), and the nucleocapsid protein (N protein) (7). Among them, the S protein is encoded by the *S* gene and plays a crucial role in PEDV invasion of host cells, mediating attachment to and fusion with the host cell membrane (8). Consequently, the *S* gene is considered a key indicator of PEDV genetic evolution. Based on this gene, PEDV can be classified into genotypes G1a, G1b, G1c (S-INDEL), G2a, G2b, and G2c (9). Structurally, the PEDV S protein is a homotrimeric glycoprotein composed of an *N*-terminal S1 subunit, which contains the receptor-binding domain, and a *C*-terminal S2 subunit, which mediates membrane fusion (10). Importantly, certain epitopes of the S protein are involved in inducing the host to produce neutralizing antibodies (11). Among them, the antigenic epitopes such as The Collagenase Equivalence, SS2, SS6, and 2C10 have been identified to elicit the production of neutralizing antibodies in the host (12–14). Given their functional significance, monitoring these important antigenic epitopes will aid in the prevention and control of PEDV infection. Nevertheless, studies have shown that these epitopes have undergone varying degrees of mutation (15). Therefore, it remains necessary to identify potential antigenic epitopes on the S protein that can induce the host cells to produce neutralizing antibodies.

In this study, an epidemiological investigation of PEDV was conducted across 20 provinces in China from 2022 to 2025. The *S* gene of the virus was sequenced, and a phylogenetic tree was constructed. Meanwhile, the changes in antigenic epitopes between the sequenced strains and the vaccine strains were comparatively analyzed. Additionally, ElliPro was used to predict antigenic epitopes located on the surface of the naturally occurring trimeric PEDV S protein. This research will provide valuable guidance for PEDV prevention and control in pig farms, as well as vaccine development.

## 2 Materials and methods

### 2.1 Collection of samples

From 2022 to 2025, a total of 2,346 samples from pigs suspected of dying from diarrhea were collected from 20 provinces in China. The samples included pig small intestinal tissues, feces, and anal swabs. The collected samples were placed in sterile 10 mL centrifuge tubes and transported to the laboratory for testing.

### 2.2 Nucleic acid extraction and detection of PEDV

Viral nucleic acids were extracted using the YALEPIC® Viral RNA Isolation Kit (YALI, Jiangsu) following the manufacturer’s instructions. Subsequently, PEDV detection was performed by RT-qPCR according to the previously established method (16). The RT-qPCR assay was carried out using the HiScript III U + One Step qRT-PCR Probe 5 × Master Mix (Vazyme, Nanjing). Negative and positive plasmid controls were included in each run. Samples with Ct value below 35 were considered positive. Primer and probe sequences are listed in Table 1.

**Table 1:** The nucleotide sequences used in this study.

Application	Name	Sequence	Length	Product size
Detection	PEDV-F	GATACTTTGGCCTCTTGTGTGT	22	150 bp
	PEDV-R	CACAACCGAATGCTATTGACG	21	
	PEDV-P	FAM-TTCAGCATCCTTATGGCTTGCATC-TAMRA	24	
Sequencing	PEDV-S1-F	AATGGTAAGTTGCTAGTGCGT	21	1972 bp
	PEDV-S1-R	GGTTTAGGCGTGCCAGTAATC	21	
	PEDV-S2-F	CTTCCTTTGGTGGTCATAGT	20	1,554 bp
	PEDV-S2-R	ATTGCTGGTTCCGCTGTAG	19	
	PEDV-S3-F	ATAGTGCGTCTCTCATCGGT	20	1,437 bp
	PEDV-S3-R	GGGCAATAAAGAACAATGAC	20	

### 2.3 Geographical analysis of PEDV infection

A geographical distribution analysis of PEDV infection was conducted. The visualization of results was performed using GraphPad Prism 10.0, Adobe Illustrator 2022, and the ggplot2 package. Geographic data were obtained from DataV (Accessed on June 11, 2025).^1^

### 2.4 Sequencing of the PEDV *S* gene

Nucleic acid samples with low Ct values from qPCR detection were selected for amplification by RT-PCR. The amplification products were analyzed using 1.5% agarose gel electrophoresis. Bands with high brightness were excised and purified, then sent to Sangon Biotech (Shanghai, China) for sequencing. The sequencing primers are listed in Table 1.

### 2.5 Sequence similarity analysis and phylogenetic tree construction of PEDV

Using the Distance method in MEGA 11.0, nucleotide and amino acid sequence similarity analyses were performed on 15 sequenced PEDV *S* genes (Supplementary Table 1). Meanwhile, together with 32 reference sequences selected from the GenBank database (Supplementary Table 3), a phylogenetic tree was constructed using the Neighbor-Joining (NJ) method in MEGA 11.0 (nucleotide sequence similarity >90%). The robustness of the phylogenetic tree was assessed by 1,000 bootstrap replicates.

### 2.6 Comparison of major antigenic epitopes of PEDV S protein

The amino acid sequences of the major antigenic epitopes COE, SS2, SS6, and 2C10 within the PEDV S protein were aligned using MegAlign software to analyze whether mutations had occurred in these epitopes.

### 2.7 Prediction of potential *N*-glycosylation sites specific to the S protein of PEDV virulent strains versus vaccine strains

Use the NetNGlyc-1.0 online server^2^ to predict potential *N*-glycosylation sites in the S proteins of the sequenced PEDV field strains and vaccine strains. Only accept results with a score > 0.5 and a Jury agreement of 9/9.

### 2.8 Prediction of antigenic epitopes in the PEDV S protein

Potential linear and discontinuous antigenic epitopes of the resolved PEDV S protein (PDB: 6VV5) were predicted using ElliPro in IEDB. Peptides with scores greater than 0.7 were selected, and PyMOL was used for the structural visualization of the PEDV S protein.

## 3 Results

### 3.1 The nucleotide sequences used in this study

### 3.2 Amplification status of the PEDV *S* gene

**Figure 1:**
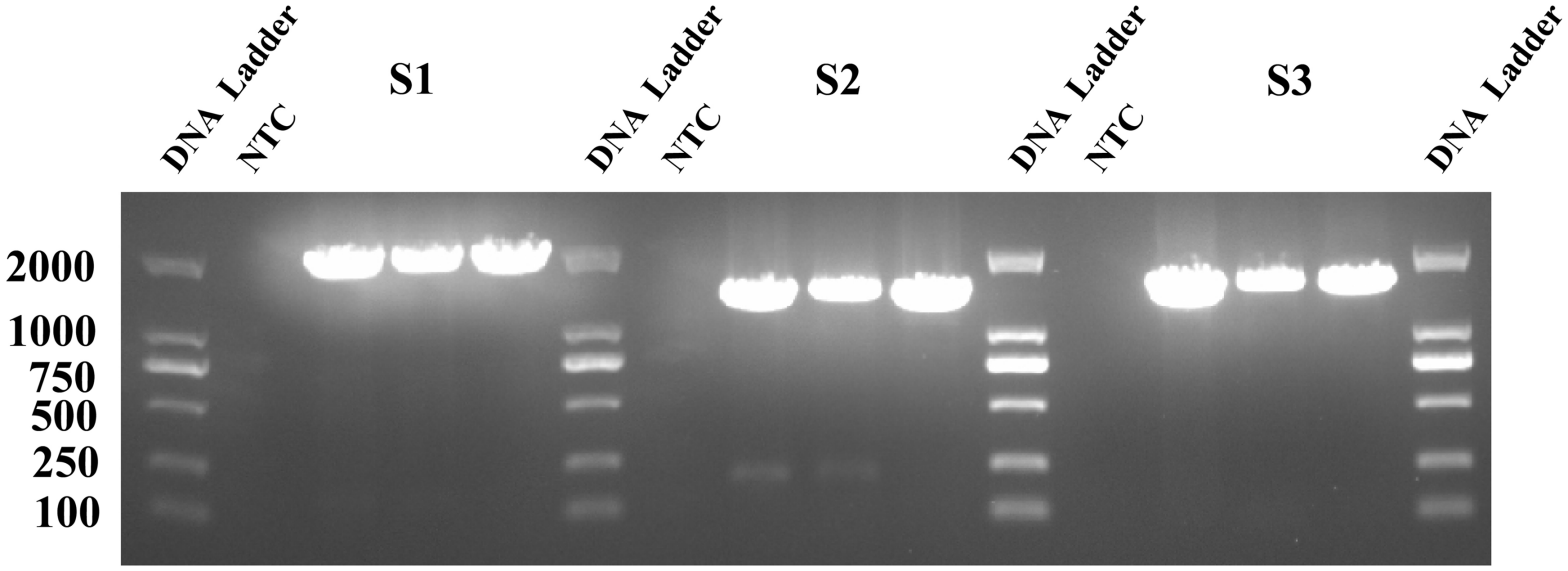
The PEDV *S* gene fragment on a 1.5% agarose gel. The nine lanes represent the corresponding amplified fragments of S1, S2, and S3, and three biological replicates were conducted.

In this study, 15 virus strains with low Ct values and representative geographic origins were selected for sequencing. The RT-PCR results are shown in Figure 1, where three clear fragments corresponding to *S1* (1972 bp), *S2* (1,554 bp), and *S3* (1,437 bp) were amplified, consistent with the expected sizes.

### 3.3 PEDV infection and geographic distribution

**Figure 2:**
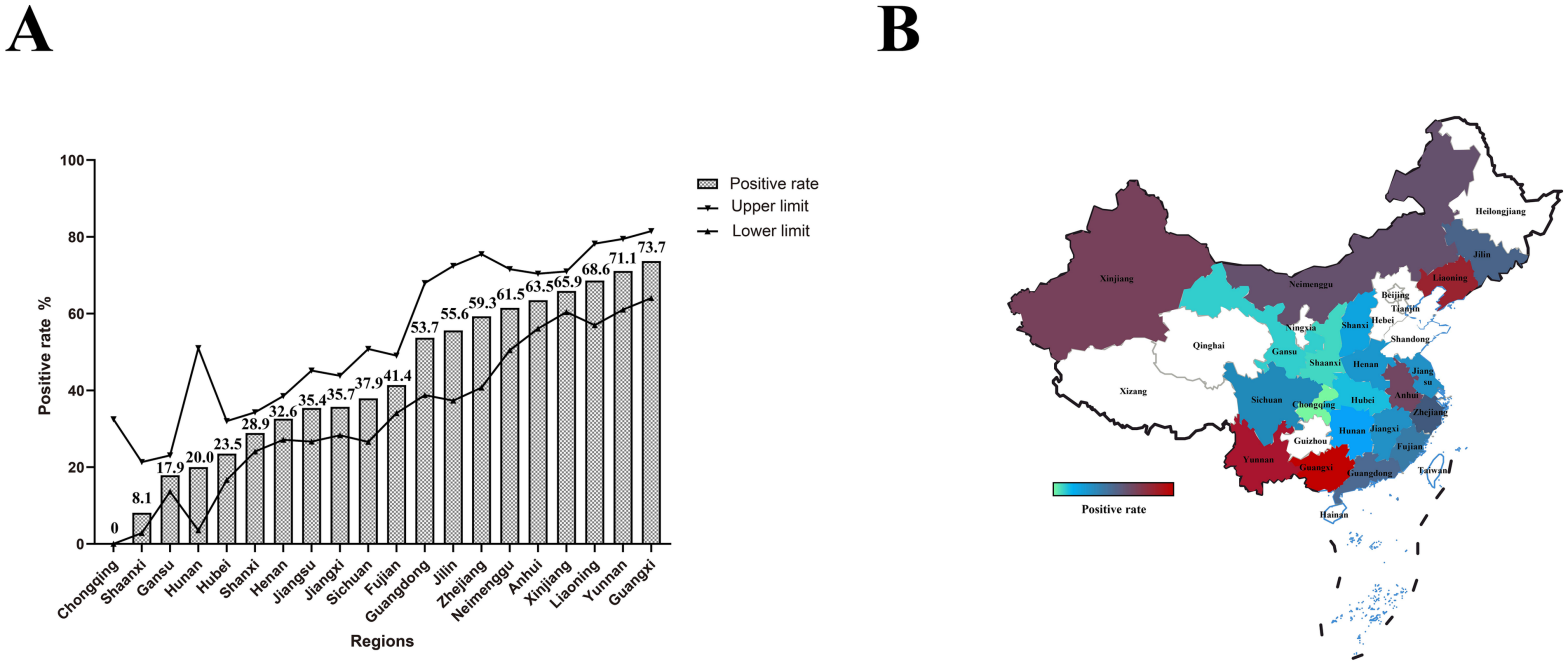
PEDV infection and geographic distribution in pigs that died of diarrhea. **(A)** The bar chart represents the positive rate of PEDV in each province, while the line chart shows the upper and lower limits of the 95% confidence interval. **(B)** Geographic distribution of PEDV. Colors ranging from green to red indicate increasing positive rates.

We conducted a survey of PEDV infection in pigs that died of diarrhea across 20 provinces in China. Among the collected samples, the positive rate of PEDV was 43.3% (95% CI: 41.3–45.3%; Figure 2A; Supplementary Table 2). Notably, the positive rates in Liaoning, Anhui, Guangxi, Inner Mongolia, Xinjiang, and Yunnan exceeded 60%. The positive rate in Shaanxi was below 10%, and no PEDV infections were detected in Chongqing. The positive rates were relatively high in Xinjiang, southern regions, and most parts of Northeast China; the coastal areas exhibited moderate positive rates, while the positive rates in inland areas were relatively low (Figure 2B).

### 3.4 Nucleotide similarity of the PEDV *S* gene and amino acid similarity of the S protein

**Figure 3:**
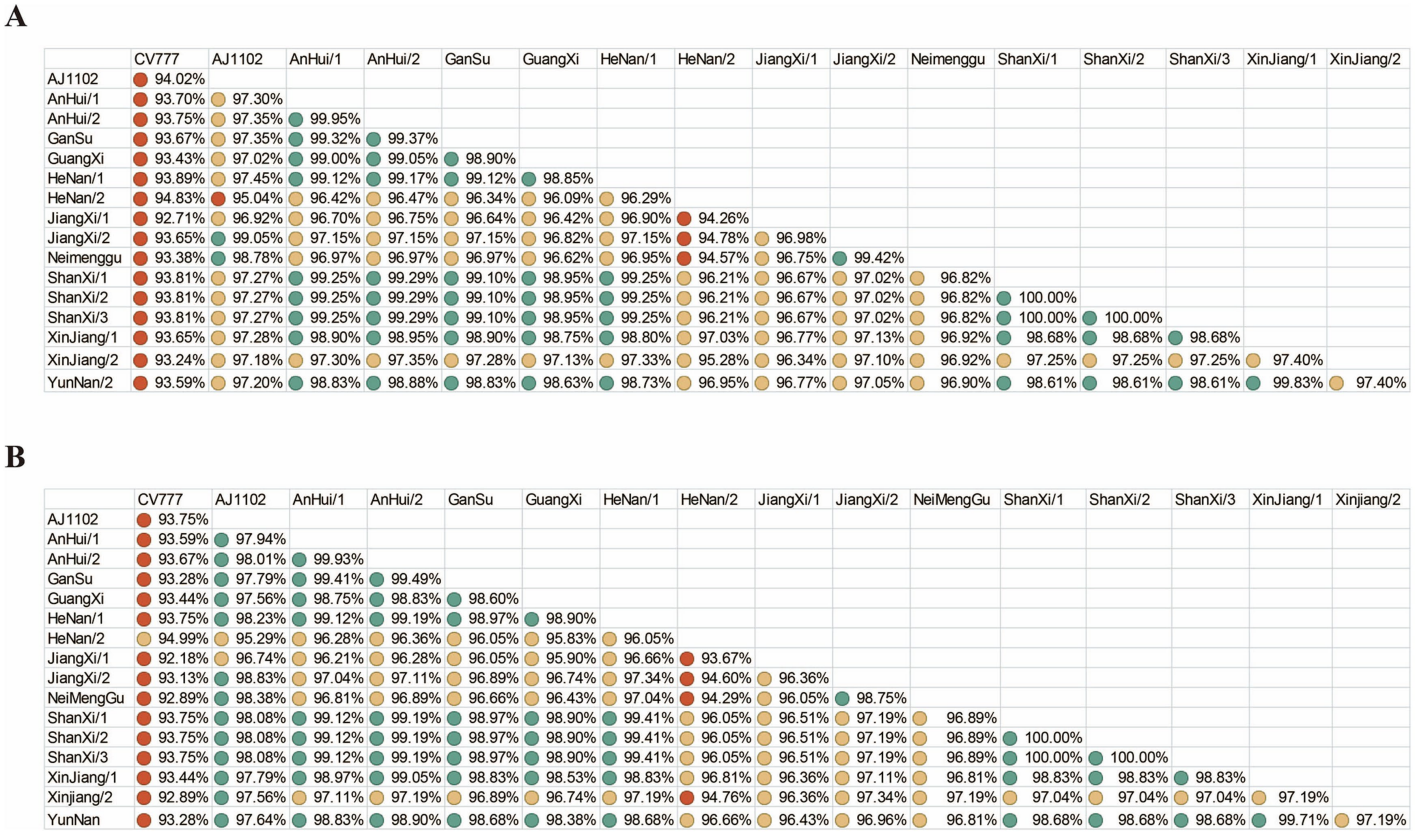
Nucleotide and amino acid similarity. **(A)** Nucleotide similarity of the PEDV *S* gene. **(B)** Amino acid similarity of the PEDV S protein. Green, yellow, and red circles represent similarity levels from high to low.

To evaluate the protective efficacy of existing vaccines, we performed nucleotide and amino acid similarity analyses between the vaccine strain CV777, AJ1102 and the sequences obtained in this study (Figure 3). The results showed that the nucleotide and amino acid similarities of the *S* gene from the 15 sequenced PEDV strains ranged from 94.26 to 100% and 93.67 to 100%, respectively. Compared with CV777, the nucleotide and amino acid similarities ranged from 92.71 to 94.83% and 92.89 to 94.99%, respectively. Compared with AJ1102, the nucleotide and amino acid similarities ranged from 95.04 to 99.05% and 95.29 to 98.83%, respectively.

### 3.5 Phylogenetic tree of PEDV

**Figure 4:**
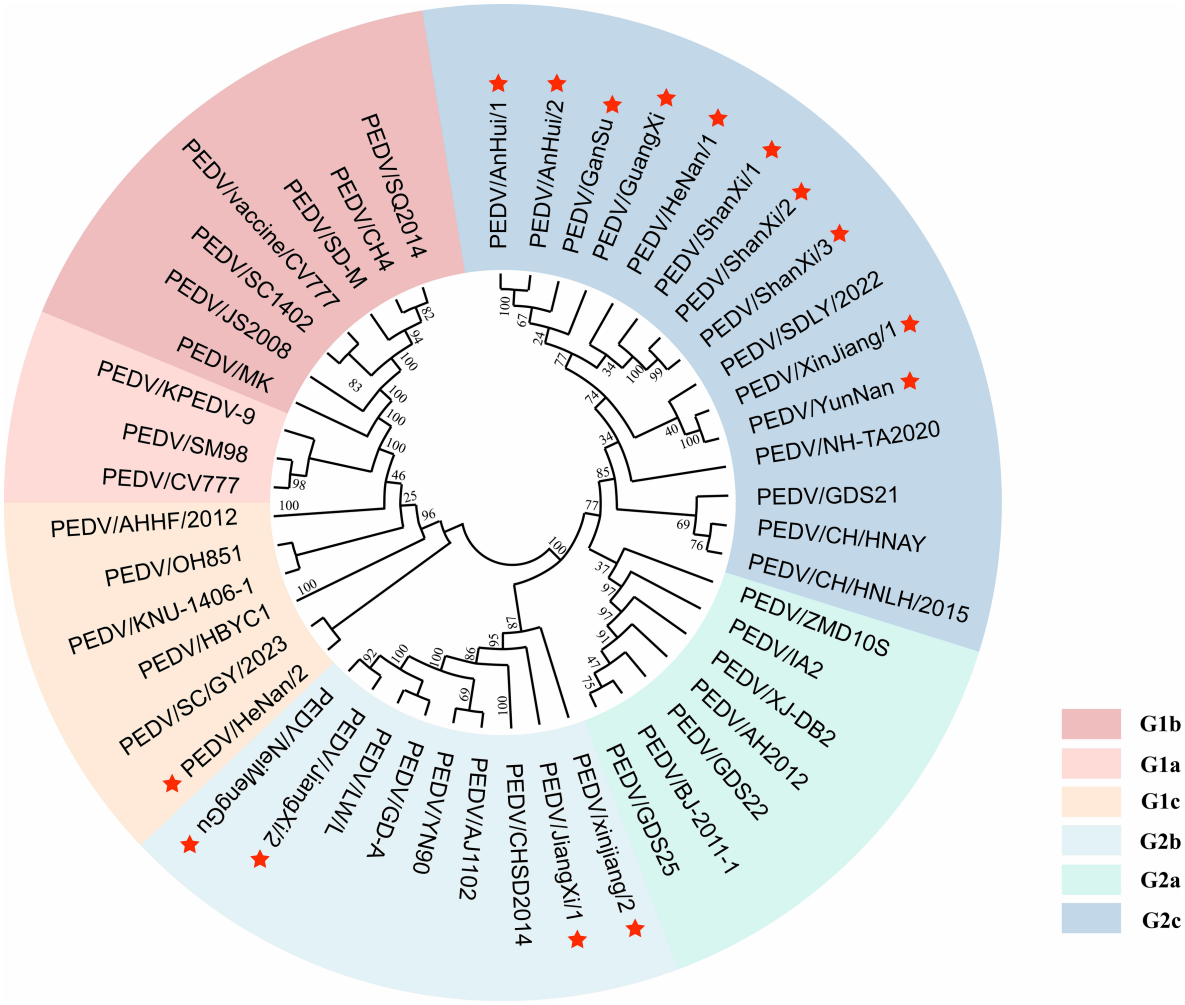
Phylogenetic tree of the PEDV *S* gene. Red pentagrams indicate the sequences obtained in this study. Different colors represent different genotypes.

A phylogenetic tree was constructed based on the *S* gene sequences of 15 sequenced PEDV strains and 32 reference strains retrieved from GenBank (Figure 4). The tree included genotypes G1a, G1b, G1c, G2a, G2b, and G2c, classified according to *S* gene typing. The results showed that among the 15 sequenced strains, 1 strain (PEDV/HeNan/2) belonged to the G1c (S-INDEL) genotype, 4 strains clustered within G2b, and the remaining 10 strains were classified as G2c, indicating that G2c is currently the predominant subtype in most regions.

### 3.6 Comparison of key antigenic epitopes of PEDV S protein

**Figure 5:**
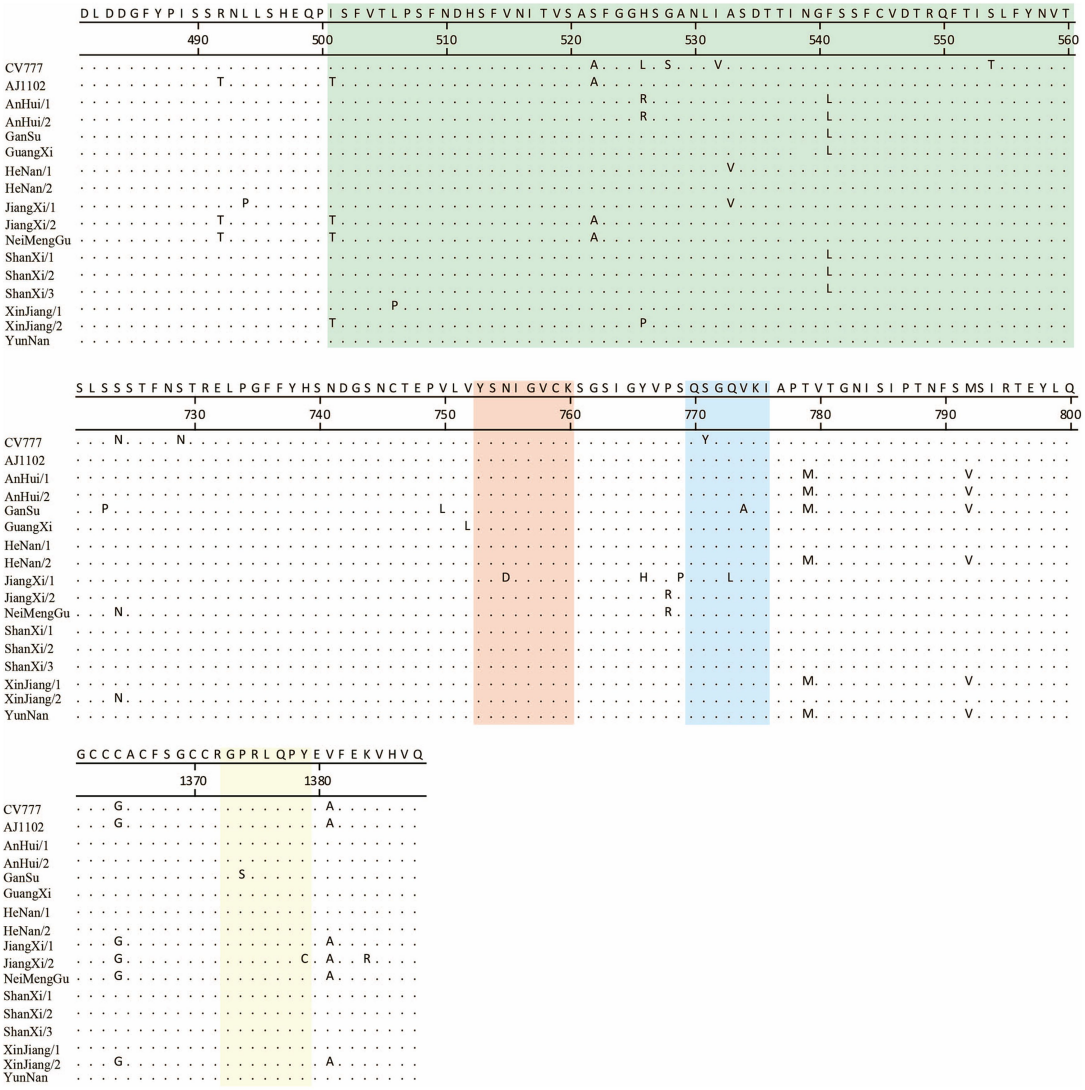
Comparison of key antigenic epitopes of the PEDV S protein. The green, orange, blue, and yellow regions correspond to the antigenic epitopes COE, SS2, SS6, and 2C10, respectively.

PEDV continues to evolve under the selective pressure of vaccines, with significant variations in its S protein. In this study, 15 newly sequenced strains were compared with the reference strains CV777 and AJ1102. Additionally, the previously identified antigenic epitopes COE, SS2, SS6, and 2C10 were analyzed (Figure 5).

The results showed that, relative to CV777, the newly sequenced strains exhibited 3 to 5 amino acid mutations in the COE region. In the SS2 epitope, a mutation (Asn^755^ to Asp^755^) was identified exclusively in the JiangXi/2 strain. Within the SS6 epitope, all newly sequenced strains shared a common amino acid substitution (Ser^771^ to Tyr^771^); furthermore, the GanSu and JiangXi/2 strains each harbored an additional mutation (Val^774^ to Ala^774^ and Gln^773^ to Leu^773^, respectively). In the 2C10 epitope, the GanSu and JiangXi/2 strains carried unique amino acid changes (Pro^1375^ to Ser^1375^ and Ile^1379^ to Cys^1379^, respectively).

When compared to AJ1102, the new strains showed 0 to 4 amino acid mutations in the COE region. The mutations observed in the SS2 epitope were consistent with those found relative to CV777. For the SS6 epitope, only the GanSu and JiangXi/2 strains exhibited amino acid substitutions (Val^774^ to Ala^774^ and Gln^773^ to Leu^773^, respectively). In the 2C10 epitope, the mutation was the same as that observed in comparison to CV777.

### 3.7 Prediction results of potential specific *N*-glycosylation sites in the S protein of 15 PEDV strains compared with the vaccine strain

**Figure 6:**
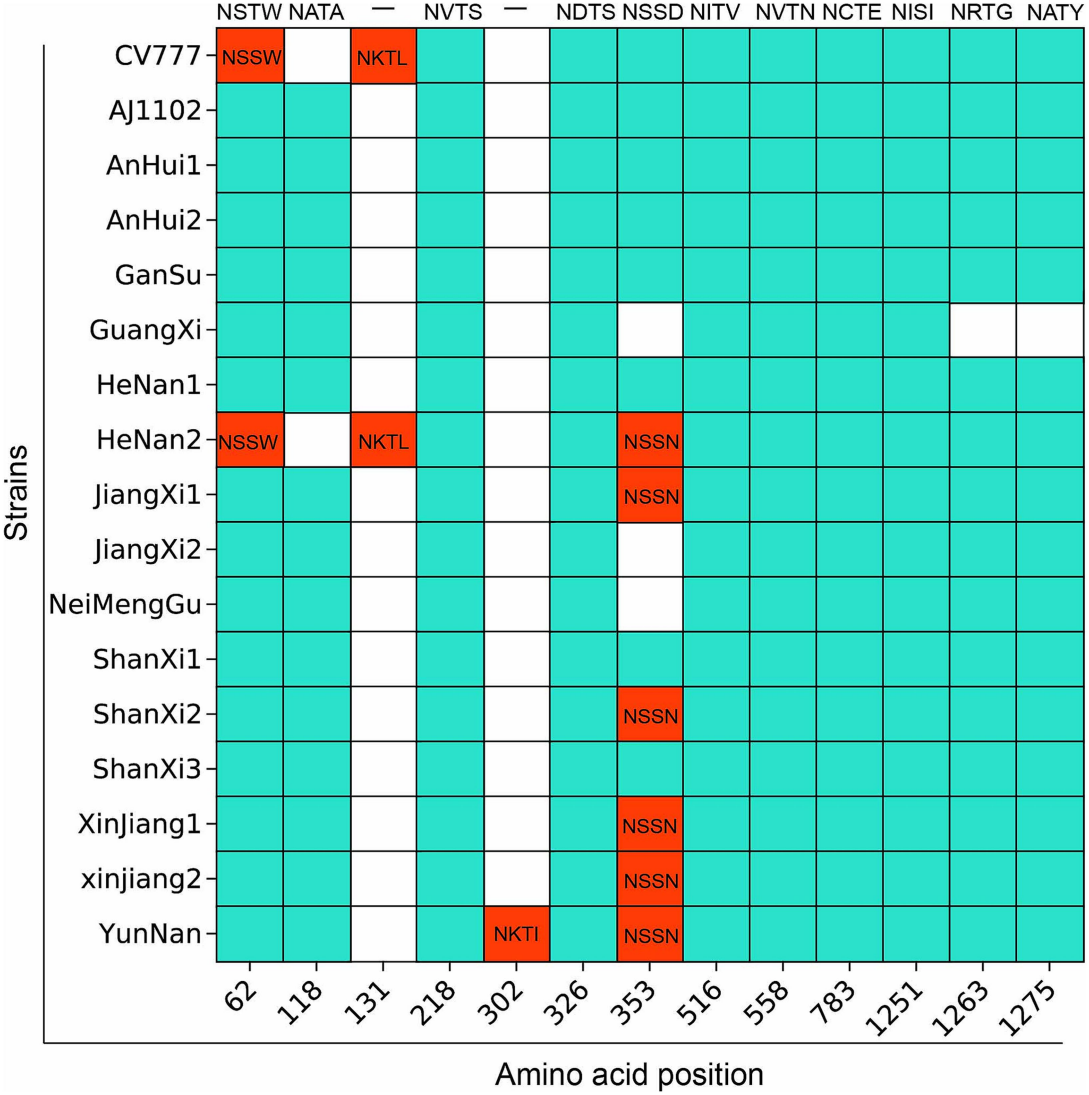
Prediction results of potential specific *N*-glycosylation sites in the S protein of 15 PEDV strains compared with the vaccine strain. The vertical axis represents the strain names, and the horizontal axis represents the amino acid sites. The blue areas indicate the same *N*-glycosylation at the same amino acid sites, while the red areas indicate differences.

Most variants at position 62 (NSTW) and position 118 (NATA) are similar to AJ1102 (Figure 6; Supplementary Table 5). However, CV777 has NSSW (Thr to Ser) at position 62 and a deletion at position 118, which may result in partial loss of protective efficacy. Notably, the PEDV/GuangXi strain, classified as G2c subtype, shows deletions of *N*-glycosylation sites at positions 353 (NSSD), 1,263 (NRTG), and 1275 (NATY) compared to AJ1102, which might lead to immune evasion. Compared to CV777, the glycosylation patterns in regions 62 and 118 differ from those of other strains, which is associated with the ability to evade traditional vaccines. The PEDV/XinJiang/2 strain exhibits a novel potential *N*-glycosylation site at position 302 (NKTI), which could alter the antigenic activity and structure of the S protein.

### 3.8 Prediction of antigenic epitopes of the PEDV S protein

**Figure 7:**
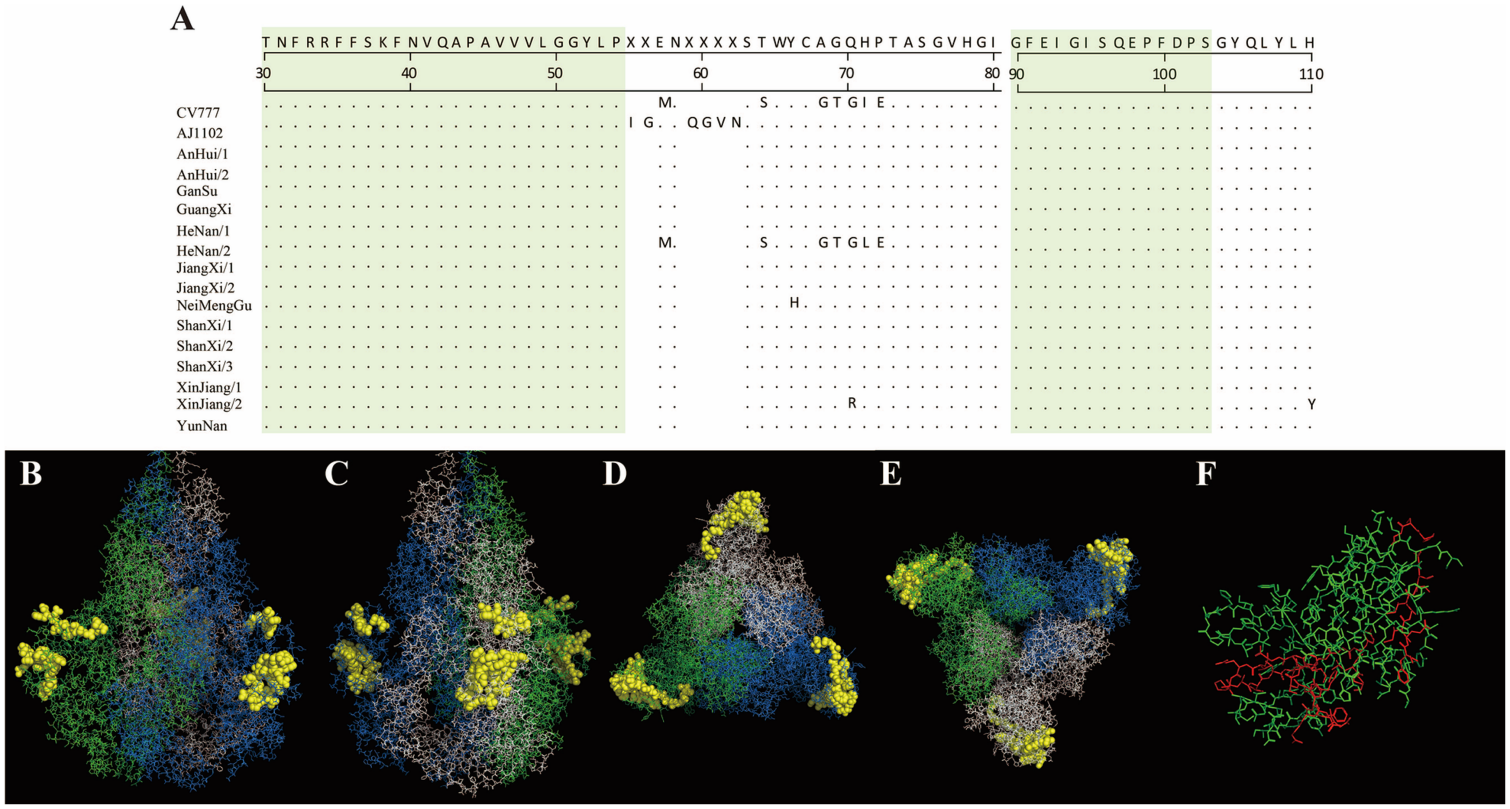
Visualization of conserved regions within continuous epitopes of the S protein in its native trimeric state. **(A)** Amino acid alignment of the sequenced strains and vaccine strains in the predicted epitope regions. **(B–E)** Visualization of conserved antigenic epitopes. The blue, green, and white regions represent the **A**, **B**, and **C** chains of the S protein trimer, respectively. Yellow spheres indicate potential antigenic epitope regions with scores greater than 0.7. **(F)** An enlarged view of the key amino acid sites of the S protein monomer, where the red areas indicate the key amino acid sites of the S protein monomer.

**Figure 8:**
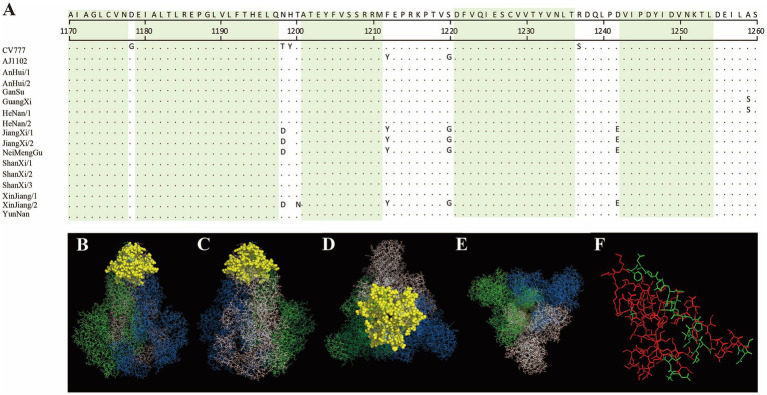
Visualization of conserved regions within continuous epitopes of the S protein in its native trimeric state. **(A)** Amino acid alignment of the sequenced strains and vaccine strains in the predicted epitope regions. **(B–E)** Visualization of conserved antigenic epitopes. The blue, green, and white regions represent the **A**, **B**, and **C** chains of the S protein trimer, respectively. Yellow spheres indicate potential antigenic epitope regions with scores greater than 0.7. **(F)** An enlarged view of the key amino acid sites of the S protein monomer, where the red areas indicate the key amino acid sites of the S protein monomer.

In this study, the antigenic epitopes of the PEDV S protein were predicted using ElliPro. Seven peptide segments with antigenicity scores greater than 0.7 were selected (Supplementary Table 4) and visualized (Supplementary Figures 1–7). Under natural conditions, the PEDV S protein exists in a trimeric state, and the structure revealed potential antigenic epitopes on the S protein in its trimeric conformation. Conserved regions among the sequenced strains and the reference strains CV777 and AJ1102 were visualized (Figures 7, 8). The potential conserved antigenic epitopes were identified in the following regions: amino acids 31–54, 90–103, 1,170–1,177, 1,179–1,197, 1,201–1,211, 1,221–1,236, and 1,243–1,254. Additionally, discontinuous (conformational) epitope prediction based on the PEDV S protein structure was performed. Two groups of discontinuous epitopes with antigenicity scores above 0.7 were selected and visualized (Supplementary Table 4; Supplementary Figures 8, 9).

## 4 Discussion

PEDV can cause diarrhea, vomiting, and dehydration in piglets, and severe cases may result in death (17). It is necessary to continuously update disease prevention and control strategies as well as vaccine development plans to effectively control and prevent PEDV infection. The primary transmission route of PEDV is fecal-oral and nasal transmission (18). From 2011 to 2014, the PEDV positive rate in 29 provinces of China ranged from approximately 61.1 to 78.5% (19). Epidemiological investigations from 2015 to 2018 showed that the positive rate of PEDV in China was approximately 66.9% (363/543) (20). From 2018 to 2021, the positive rate of PEDV in the southern region of China was 55.8% (3,986/7107) (21). Epidemiological data from seven regions in China between 2020 and 2022 showed that the positive rate of PEDV was approximately 57.4% (199/347) (22). This study investigated the PEDV positive rate in 20 provinces of China from 2022 to 2025, which was 43.3% (1,015/2346). Regions with high positive rates were mainly concentrated in southern and northeastern China, while regions with low positive rates were primarily located in the western and northern parts of the country. These areas may have maintained lower positive rates due to factors such as lower pig farm density, geographical isolation, limited mobility, or effective prevention and control measures.

The similarity results of the PEDV *S* gene and amino acids compared with CV777 and AJ1102 indicate a significant difference from CV777, suggesting that the vaccine’s protective efficacy may be weakened. In contrast, there is a relatively high similarity with AJ1102, but a certain degree of mutation has also occurred, which may affect the efficacy of the vaccine in future evolution.

The S protein of PEDV contains numerous antigenic epitopes that can induce the host to produce neutralizing antibodies, and most of these epitopes are located in the S1 region (23, 24). Under the selective pressure exerted by vaccines, mutations occur in the PEDV S protein (25). When mutations occur in the antigenic epitopes on the S protein, PEDV’s ability to bind antibodies is significantly reduced (26). Therefore, monitoring whether antigenic epitopes on the PEDV S protein undergo changes is of great significance for the prevention and control of PEDV and the development of vaccines. Previous studies have identified several important antigenic epitopes. The Core Neutralizing Epitope (COE), combined with an adjuvant and administered into mice, can induce the production of a large amount of PEDV neutralizing antibodies (27, 28). A PEDV vaccine based on the COE antigenic epitope has already been developed (27). In addition, the SS2, SS6, and 2C10 epitopes have also been identified (29, 30).

This study analyzed the previously identified COE, SS2, SS6, and 2C10 epitopes, finding that the newly sequenced strains exhibit varying degrees of mutations in these epitope regions. These mutations may potentially affect the protective efficacy of current mainstream vaccines, indicating the need for further monitoring.

The extensive *N*-glycosylation of the S protein is a prerequisite for the virus to enter host cells, complete membrane fusion, and carry out subsequent replication. An intact *N*-glycosylation pathway is essential for PEDV proliferation (31). The addition, loss, or positional changes of *N*-glycosylation sites can create a glycan shield on the surface of the S protein, masking neutralizing epitopes and thereby helping the virus evade host antibody recognition. The GII subtype (such as GIIc) has acquired new glycosylation sites (N62, N118) in the Domain 0 region of the S protein, which is believed to be associated with its high pathogenicity and ability to escape traditional vaccines (32). Therefore, monitoring the *N*-glycosylation sites of the PEDV S protein is also of certain significance for future vaccine development.

Linear and discontinuous epitopes hold significant importance in immunology. Linear antigen epitopes are composed of consecutively arranged amino acids and generally do not depend on the three-dimensional structure of the antigen. B cells can recognize linear epitopes exposed on the surface of antigens and, upon activation, produce specific antibodies targeting these epitopes. Discontinuous epitopes, which are also one of the main types of epitopes recognized by B cells, consist of residues that are brought into close proximity through the spatial folding of the protein, forming a specific three-dimensional structure that is recognized by antibodies or B cell receptors (33).

ElliPro is an online tool that can be used to identify antigenic epitopes in structural proteins. ElliPro is considered a potentially useful research tool for identifying antigens epitopes (34). Previously, researchers used ElliPro to predict linear B-cell epitopes on the envelope protein of the Zika virus (35). In 2023, 24 linear epitopes and 7 discontinuous epitopes of avian-origin *Escherichia coli* were predicted using ElliPro, and two of these peptide segments were selected as candidate peptides (36). In addition, other researchers used the IEDB ElliPro server to predict discontinuous and linear epitope-specific B-cell epitopes of the SARS-CoV-2 spike protein (37).

This study used ElliPro to predict 7 linear epitopes and 2 discontinuous epitopes of the PEDV S protein in its trimeric state, which has already been resolved. Conserved epitopes were identified among those with high scores. However, these antigenic epitopes based on bioinformatics analysis still require further validation through *in vitro* and *in vivo* experiments.

## 5 Conclusion

In summary, this study revealed that among 2,346 pig samples from pigs that died of diarrhea collected across 20 provinces in China during 2022–2025, the PEDV positive rate was 43.3% (1,015/2,346). Three subgroups were identified, among which 1 strains belonged to G1c, 4 strains to G2b, and the remaining 10 strains to G2c, indicating that G2c is currently the dominant subtype. The nucleotide and amino acid similarities between the sequenced strains and the CV777 strain ranged from 92.71 to 94.83% and 92.89 to 94.99%, respectively. Mutations of varying degrees were observed in the neutralizing epitopes COE, SS2, SS6, and 2C10. Prediction of antigenic epitopes identified aa31–54, 90–103, 1,170–1,177, 1,179–1,197, 1,201–1,211, 1,221–1,236, and 1,243–1,254 as conserved potential antigenic regions. These findings provide important references and insights for developing effec-tive PED control strategies, as well as for the research and development of vaccines and diagnostic methods.

## Data Availability

The original contributions presented in the study are included in the article/Supplementary material, further inquiries can be directed to the corresponding author/s.

## Footnotes

^1^ https://datav.aliyun.com/portal/school/atlas/area_selector

^2^ https://services.healthtech.dtu.dk/services/NetNGlyc-1.0/
