# Supplementary material for: Molecular characteristics and potential antigenic epitope analysis of porcine epidemic diarrhea virus in China from 2022 to 2025
Source: Front Vet Sci. 2025 Oct 8;12:1667063. doi: 10.3389/fvets.2025.1667063 (PMC12542869; doi:10.3389/fvets.2025.1667063)
Supplement: Supplementary file 1 [file Data_Sheet_1.docx]

Supplementary Material

**Supplementary Tables and Figures Legends**

**Supplementary Table 1. The information of PEDV strains sequenced in this study.**

**Supplementary Table 2. Information regarding PEDV in samples from diarrhea-suspected deceased animals in this study.**

**Supplementary Table 3. PEDV *S* gene sequences and their GenBank accession numbers used for comparison.**

**Supplementary Table 4. ElliPro predicted the linear and discontinuous antigenic epitopes on the surface of the PEDV S protein.**

**Supplementary Table 5.** **Prediction results of potential specific *N*-glycosylation sites in the S protein of 15 PEDV strains compared with the vaccine strain.**

**Supplementary Figure 1-7.** **Visualization of linear epitopes of the S protein in its native trimeric state predicted by ElliPro.**

A-D. The blue, green, and white regions represent the A, B, and C chains of the S protein trimer, respectively. Yellow spheres indicate potential antigenic epitope regions with scores greater than 0.7. E. An enlarged view of the key amino acid sites of the S protein monomer, where the red areas indicate the key amino acid sites of the S protein monomer.

**Supplementary Figure 8-9.** **Visualization of discontinuous epitopes of the S protein in its native trimeric state predicted by ElliPro.**

A-D. The blue, green, and white regions represent the A, B, and C chains of the S protein trimer, respectively. Yellow spheres indicate potential antigenic epitope regions with scores greater than 0.7 E. An enlarged view of the key amino acid sites of the S protein monomer, where the red areas indicate the key amino acid sites of the S protein monomer.

**Supplementary Table 1. The information of PEDV strains sequenced in this study.**

| **Strains** | **Collection data** | **Location** | **Accession number** |
| --- | --- | --- | --- |
| PEDV/AnHui/1 | Oct-2023 | AnHui | PV844362 |
| PEDV/AnHui/2 | Jul-2024 | AnHui | PV844363 |
| PEDV/GanSu | Jan-2025 | GanSu | PV844364 |
| PEDV/GuangXi | Dec-2024 | GuangXi | PV844365 |
| PEDV/HeNan/1 | Feb-2025 | HeNan | PV844366 |
| PEDV/HeNan/2 | May-2025 | HeNan | PV844367 |
| PEDV/JiangXi/1 | Oct-2024 | JiangXi | PV844368 |
| PEDV/JiangXi/2 | Sep-2023 | JiangXi | PV844369 |
| PEDV/Neimenggu | Sep-2024 | Neimenggu | PV844370 |
| PEDV/ShanXi/1 | Aug-2023 | ShanXi | PV844371 |
| PEDV/ShanXi/2 | Mar-2025 | ShanXi | PV844372 |
| PEDV/ShanXi/3 | Dec-2024 | ShanXi | PV844373 |
| PEDV/XinJiang/1 | Jan-2024 | XinJiang | PV844374 |
| PEDV/XinJiang/2 | May-2024 | XinJiang | PV844375 |
| PEDV/YunNan | Apr-2024 | YunNan | PV844376 |

**Supplementary Table 2. Information regarding PEDV in samples from diarrhea-suspected deceased animals in this study.**

| **Regions** | **Number of samples** | **Number of positives** | **Positive rate** | **95% CI** |
| --- | --- | --- | --- | --- |
| Chongqing | 8 | 0 | 0 | [0-32.4%] |
| Shaanxi | 37 | 3 | 8.1% | [2.8%-21.3%] |
| Gansu | 252 | 45 | 17.9% | [13.6%-23.1%] |
| Hunan | 10 | 2 | 20% | [5.7%-51%] |
| Hubei | 115 | 27 | 23.5% | [16.7%-32%] |
| Shanxi | 301 | 87 | 28.9% | [24.1%-34.3%] |
| Henan | 258 | 84 | 32.6% | [27.1%-38.5%] |
| Jiangsu | 99 | 35 | 35.4% | [26.6%-45.2%] |
| Jiangxi | 143 | 51 | 35.7% | [28.3%-43.8%] |
| Sichuan | 58 | 22 | 37.9% | [26.6%-50.8%] |
| Fujian | 162 | 67 | 41.4% | [34.1%-49.1%] |
| Guangdong | 41 | 22 | 53.7% | [38.7%-67.9%] |
| Jilin | 27 | 15 | 55.6% | [37.3%-72.4%] |
| Zhejiang | 27 | 16 | 59.3% | [40.7%-75.5%] |
| Neimenggu | 78 | 48 | 61.5% | [50.4%-71.5%] |
| Anhui | 170 | 108 | 63.5%  3.65.9% | [56.1%-70.4%] |
| Xinjiang | 305 | 201 | 65.9% | [60.4%-71%] |
| Liaoning | 70 | 48 | 68.6% | [57%-78.2%] |
| Yunnan | 90 | 64 | 71.1% | [61%-79.5%] |
| Guangxi | 95  234 | 70 | 73.7% | [64%-81.5%] |
| Total | 2346 | 1015 | 43.3% | [41.3%-45.3%] |

**Supplementary Table 3. PEDV *S* gene sequences and their GenBank accession numbers used for comparison**

| **Sequence Name** | **Sequence number (GenBank)** |
| --- | --- |
| PEDV/CV777 | AF353511 |
| PEDV/KPEDV-9 | KF898124 |
| PEDV/SM98 | GU937797 |
| PEDV/JS2008 | KC210146 |
| PEDV/MK | AB548624 |
| PEDV/SD-M | JX560761 |
| PEDV/vaccine/CV777 | KT323979 |
| PEDV/SC1402 | KP162057 |
| PEDV/CH4 | JQ239432 |
| PEDV/SQ2014 | KP728470 |
| PEDV/BJ-2011-1 | JN825712 |
| PEDV/XJ-DB2 | KM287429 |
| PEDV/GDS22 | MH726368 |
| PEDV/ZMD10S | KY211073 |
| PEDV/IA2 | KF468754 |
| PEDV/GDS25 | MH726365 |
| PEDV/AH2012 | KC210145 |
| PEDV/AJ1102 | JX188454 |
| PEDV/LW/L | MK392335 |
| PEDV/GD-A | JX112709 |
| PEDV/YN90 | KT021231. |
| PEDV/CHSD2014 | KX791060 |
| PEDV/SDLY/2022 | OQ122089 |
| PEDV/NH-TA2020 | ON168803 |
| PEDV/GDS21 | MH726371 |
| PEDV/CH/HNLH/2015 | KT199103 |
| PEDV/CH/HNLH | KR809885 |
| PEDV/OH851 | KJ399978 |
| PEDV/HBYC1 | KY775055 |
| PEDV/KNU-1406-1 | KM403155 |
| PEDV/AHHF/2012 | JX018181 |
| PEDV/SC/GY/2023 | PQ507873 |

**Supplementary Table 4. ElliPro predicted the linear and discontinuous antigenic epitopes on the surface of the PEDV S protein.**

| **Number** | **Chains** | **Start** | **End** | **Amino acids of linear epitopes** | **Number of Residues** | **Score** | **3D visualization location** |
| --- | --- | --- | --- | --- | --- | --- | --- |
| **1** | **A/B/C** | **31** | **103** | **NFRRFFSKFNVQAPAVVVLGGYLPIVHGIFVSGFEIGISQPS** | **42** | **0.863** | **Supplementary Fig 1** |
| **2** | **A/B/C** | **186** | **260** | **KNDWSRVATAMQYVYEPTYYMLNVTSAGEDGISYQPCTANCIGYAANVFATEPNGHIPEGFSFNNWF** | **67** | **0.818** | **Supplementary Fig 2** |
| **3** | **A/B/C** | **1160** | **1254** | **PSDFVDVIAIAGLCVNDEIALTLREPGLVLFTHEEYFVSSRRMFEPRKPTVSDFVQIESCVVTYVNLTRDQLPDVIPDYIDVNKTLDE** | **88** | **0.817** | **Supplementary Fig 3** |
| **4** | **A/B/C** | **280** | **410** | **LVNCLLAIPKIYQFFSFNQTIDGVCNGAAVQRAPEALRFNINDISVILASIVLHTALGTNFSFVCSNSSNPHLATPYYCFLKVDTYNSTVYKFLAVLPPTVREIVITKYGDVYVNG** | **116** | **0.764** | **Supplementary Fig 4** |
| **5** | **A/B/C** | **153** | **183** | **AIPAHVVGITWDNDRVTVFSDKIYYF** | **26** | **0.726** | **Supplementary Fig 5** |
| **6** | **A/B/C** | **413** | **445** | **YLHLGLLDAVTINFTGFWTIASTN** | **24** | **0.705** | **Supplementary Fig 6** |
| **7** | **A/B/C** | **105** | **125** | **YQLYLHKATTARLRI** | **15** | **0.703** | **Supplementary Fig 7** |
|  | | | | **Amino acids of** **discontinuous epitopes** |  | | |
| **1** |  |  |  | **A:N31, A:F32, A:R33, A:R34, A:F35, A:F36, A:S37, A:K38, A:F39, A:N40, A:V41, A:Q42, A:A43, A:P44, A:A45, A:V46, A:V47, A:V48, A:L49, A:G50, A:G51, A:Y52, A:L53, A:P54, A:I55, A:V77, A:H78, A:G79, A:I80, A:F81, A:V82, A:S83, A:G90, A:F91, A:E92, A:I93, A:G94, A:I95, A:S96, A:Q97, A:P102, A:S103, A:G104, A:Y105, A:Q106, A:L107, A:Y108, A:L109, A:H110, A:K111, A:A112, A:T113, A:T120, A:A121, A:R122, A:L123, A:R124, A:I125, A:F128, A:P129, A:S130, A:A153, A:I154, A:P155, A:A156, A:H157, A:V163, A:V164, A:G165, A:I166, A:T167, A:W168, A:D169, A:N170, A:D171, A:R172, A:V173, A:T174, A:V175, A:F176, A:S177, A:D178, A:K179, A:I180, A:Y181, A:Y182, A:F183, A:F185, A:K186, A:N187, A:D188, A:W189, A:V192, A:A193, A:T194, A:A203, A:M204, A:Q205, A:Y206, A:V207, A:Y208, A:E209, A:P210, A:T211, A:Y212, A:Y213, A:M214, A:L215, A:N216, A:V217, A:T218, A:S219, A:A220, A:G221, A:E222, A:D223, A:G224, A:I225, A:S226, A:Y227, A:Q228, A:P229, A:C230, A:T231, A:A232, A:N233, A:C234, A:I235, A:G236, A:Y237, A:A238, A:A239, A:N240, A:V241, A:F242, A:A243, A:T244, A:E245, A:P246, A:N247, A:G248, A:H249, A:I250, A:P251, A:E252, A:G253, A:F254, A:S255, A:F256, A:N257, A:N258, A:W259, A:F260, A:L280, A:V281, A:N282, A:C283, A:L284, A:L285, A:A286, A:I287, A:P288, A:K289, A:I290, A:Y291, A:Q295, A:F296, A:F297, A:S298, A:F299, A:N300, A:Q301, A:T302, A:I303, A:D304, A:G305, A:V306, A:C307, A:N308, A:G309, A:A310, A:A311, A:V312, A:Q313, A:R314, A:A315, A:P316, A:E317, A:A318, A:L319, A:R320, A:F321, A:N322, A:I323, A:N324, A:D325, A:I326, A:S327, A:V328, A:I329, A:L330, A:A331, A:S334, A:I335, A:V336, A:L337, A:H338, A:T339, A:A340, A:L341, A:G342, A:T343, A:N344, A:F345, A:S346, A:F347, A:V348, A:C349, A:S350, A:N351, A:S352, A:S353, A:N354, A:P355, A:H356, A:L357, A:A358, A:T359, A:P370, A:Y371, A:Y372, A:C373, A:F374, A:L375, A:K376, A:V377, A:D378, A:T379, A:Y380, A:N381, A:S382, A:T383, A:V384, A:Y385, A:K386, A:F387, A:L388, A:A389, A:V390, A:L391, A:P392, A:P393, A:T394, A:V395, A:R396, A:E397, A:I398, A:V399, A:I400, A:K402, A:Y403,** | **374** | **0.748** | **Supplementary Fig 8** |
| **2** |  |  |  | **A:I774, A:A775, A:P776, A:T777, A:V778, A:T779, A:G780, A:N781, A:I782, A:S783, A:I784, A:P785, A:T786, A:N787, A:F788, A:Q860, A:L861, A:A862, A:T863, A:I864, A:S865, A:S866, A:F867, A:N868, A:G869, A:D870, A:G871, A:Y872, A:N873, A:F874, A:T875, A:N876, A:L878, A:G879, A:G964, A:G965, A:F966, A:T967, A:S968, A:A969, A:A970, A:A971, A:L972, A:P973, A:S975, A:Y976, A:Q979, A:N983, A:A986, A:L987, A:Q988, A:T989, A:D990, A:V991, A:L992, A:Q993, A:R994, A:N995, A:Q996, A:Q997, A:L998, A:L999, A:A1000, A:E1001, A:N1004, A:P1160, A:S1161, A:D1162, A:F1163, A:V1164, A:D1165, A:V1166, A:I1167, A:A1168, A:I1169, A:A1170, A:G1171, A:L1172, A:C1173, A:V1174, A:N1175, A:D1176, A:E1177, A:I1178, A:A1179, A:L1180, A:T1181, A:L1182, A:R1183, A:E1184, A:P1185, A:G1186, A:L1187, A:V1188, A:L1189, A:F1190, A:T1191, A:H1192, A:E1193, A:E1201, A:Y1202, A:F1203, A:V1204, A:S1205, A:S1206, A:R1207, A:R1208, A:M1209, A:F1210, A:E1211, A:P1212, A:R1213, A:K1214, A:P1215, A:T1216, A:V1217, A:S1218, A:D1219, A:F1220, A:V1221, A:Q1222, A:I1223, A:E1224, A:S1225, A:C1226, A:V1227, A:V1228, A:T1229, A:Y1230, A:V1231, A:N1232, A:L1233, A:T1234, A:R1235, A:D1236, A:Q1237, A:L1238, A:P1239, A:D1240, A:V1241, A:I1242, A:P1243, A:D1244, A:Y1245, A:I1246, A:D1247, A:V1248, A:N1249, A:K1250, A:T1251, A:L1252, A:D1253, A:E1254, B:I774, B:A775, B:P776, B:T777, B:V778, B:T779, B:G780, B:N781, B:I782, B:S783, B:I784, B:P785, B:T786, B:N787, B:F788, B:Q860, B:L861, B:A862, B:T863, B:I864, B:S865, B:S866, B:F867, B:N868, B:G869, B:D870, B:G871, B:Y872, B:N873, B:F874, B:T875, B:N876, B:L878, B:G879, B:G964, B:G965, B:F966, B:T967, B:S968, B:A969, B:A970, B:A971, B:L972, B:P973, B:S975, B:Y976, B:Q979, B:N983, B:A986, B:L987, B:Q988, B:T989, B:D990, B:V991, B:L992, B:Q993, B:R994, B:N995, B:Q996, B:Q997, B:L998, B:L999, B:A1000, B:E1001, B:N1004, B:P1160, B:S1161, B:D1162, B:F1163, B:V1164, B:D1165, B:V1166, B:I1167, B:A1168, B:I1169, B:A1170, B:G1171, B:L1172, B:C1173, B:V1174, B:N1175, B:D1176, B:E1177, B:I1178, B:A1179, B:L1180, B:T1181, B:L1182, B:R1183, B:E1184, B:P1185, B:G1186, B:L1187, B:V1188, B:L1189, B:F1190, B:T1191, B:H1192, B:E1193, B:E1201, B:Y1202, B:F1203, B:V1204, B:S1205, B:S1206, B:R1207, B:R1208, B:M1209, B:F1210, B:E1211, B:P1212, B:R1213, B:K1214, B:P1215, B:T1216, B:V1217, B:S1218, B:D1219, B:F1220, B:V1221, B:Q1222, B:I1223, B:E1224, B:S1225, B:C1226, B:V1227, B:V1228, B:T1229, B:Y1230, B:V1231, B:N1232, B:L1233, B:T1234, B:R1235, B:D1236, B:Q1237, B:L1238, B:P1239, B:D1240, B:V1241, B:I1242, B:P1243, B:D1244, B:Y1245, B:I1246, B:D1247, B:V1248, B:N1249, B:K1250, B:T1251, B:L1252, B:D1253, B:E1254, C:I774, C:A775, C:P776, C:T777, C:V778, C:T779, C:G780, C:N781, C:I782, C:S783, C:I784, C:P785, C:T786, C:N787, C:F788, C:Q860, C:L861, C:A862, C:T863, C:I864, C:S865, C:S866, C:F867, C:N868, C:G869, C:D870, C:G871, C:Y872, C:N873, C:F874, C:T875, C:N876, C:L878, C:G879, C:G964, C:G965, C:F966, C:T967, C:S968, C:A969, C:A970, C:A971, C:L972, C:P973, C:S975, C:Y976, C:Q979, C:N983, C:A986, C:L987, C:Q988, C:T989, C:D990, C:V991, C:L992, C:Q993, C:R994, C:N995, C:Q996, C:Q997, C:L998, C:L999, C:A1000, C:E1001, C:N1004, C:P1160, C:S1161, C:D1162, C:F1163, C:V1164, C:D1165, C:V1166, C:I1167, C:A1168, C:I1169, C:A1170, C:G1171, C:L1172, C:C1173, C:V1174, C:N1175, C:D1176, C:E1177, C:I1178, C:A1179, C:L1180, C:T1181, C:L1182, C:R1183, C:E1184, C:P1185, C:G1186, C:L1187, C:V1188, C:L1189, C:F1190, C:T1191, C:H1192, C:E1193, C:E1201, C:Y1202, C:F1203, C:V1204, C:S1205, C:S1206, C:R1207, C:R1208, C:M1209, C:F1210, C:E1211, C:P1212, C:R1213, C:K1214, C:P1215, C:T1216, C:V1217, C:S1218, C:D1219, C:F1220, C:V1221, C:Q1222, C:I1223, C:E1224, C:S1225, C:C1226, C:V1227, C:V1228, C:T1229, C:Y1230, C:V1231, C:N1232, C:L1233, C:T1234, C:R1235, C:D1236, C:Q1237, C:L1238, C:P1239, C:D1240, C:V1241, C:I1242, C:P1243, C:D1244, C:Y1245, C:I1246, C:D1247, C:V1248, C:N1249, C:K1250, C:T1251, C:L1252, C:D1253, C:E1254** | **489** | **0.728** | **Supplementary Fig 9** |

| **Strains** |  | | **High-specificity *N*-glycosylation sites of S protein** | | | | | | | | | | | | | |
| --- | --- | --- | --- | --- | --- | --- | --- | --- | --- | --- | --- | --- | --- | --- | --- | --- |
|  | **62** | **118** |  | **131** | **218** | **302** | **326** | **353** | **516** |  | **558** | **745** | **783** | **1251** | **1263** | **1275** |
| PEDV/CV777 | NSSW | － |  | NKTL | NVTS | － | NDTS | NSSD | NITV |  | NVTN | NCTE | NISI | NKTL | NRTG | NATY |
| PEDV/AJ1102 | NSTW | NATA |  | － | NVTS | － | NDTS | NSSD | NITV |  | NVTN | NCTE | NISI | NKTL | NRTG | NATY |
| PEDV/AnHui/1 | NSTW | NATA |  | － | NVTS | － | NDTS | NSSD | NITV |  | NVTN | NCTE | NISI | NKTL | NRTG | NATY |
| PEDV/AnHui/2 | NSTW | NATA |  | － | NVTS | － | NDTS | NSSD | NITV |  | NVTN | NCTE | NISI | NKTL | NRTG | NATY |
| PEDV/GanSu | NSTW | NATA |  | － | NVTS | － | NDTS | NSSD | NITV |  | NVTN | NCTE | NISI | NKTL | NRTG | NATY |
| PEDV/GuangXi | NSTW | NATA |  | － | NVTS | － | NDTS | － | NITV |  | NVTN | NCTE | NISI | NKTL | － | － |
| PEDV/HeNan/1 | NSTW | NATA |  | － | NVTS | － | NDTS | NSSD | NITV |  | NVTN | NCTE | NISI | NKTL | NRTG | NATY |
| PEDV/HeNan/2 | NSSW | － |  | NKTL | NVTS | － | NDTS | NSSN | NITV |  | NVTN | NCTE | NISI | NKTL | NRTG | NATY |
| PEDV/JiangXi/1 | NSTW | NATA |  | － | NVTS | － | NDTS | NSSN | NITV |  | NVTN | NCTE | NISI | NKTL | NRTG | NATY |
| PEDV/JiangXi/2 | NSTW | NATA |  | － | NVTS | － | NDTS | － | NITV |  | NVTN | NCTE | NISI | NKTL | NRTG | NATY |
| PEDV/Neimenggu | NSTW | NATA |  | － | NVTS | － | NDTS | － | NITV |  | NVTN | NCTE | NISI | NKTL | NRTG | NATY |
| PEDV/ShanXi/1 | NSTW | NATA |  | － | NVTS | － | NDTS | NSSD | NITV |  | NVTN | NCTE | NISI | NKTL | NRTG | NATY |
| PEDV/ShanXi/2 | NSTW | NATA |  | － | NVTS | － | NDTS | NSSD | NITV |  | NVTN | NCTE | NISI | NKTL | NRTG | NATY |
| PEDV/ShanXi/3 | NSTW | NATA |  | － | NVTS | － | NDTS | NSSD | NITV |  | NVTN | NCTE | NISI | NKTL | NRTG | NATY |
| PEDV/XinJiang/1 | NSTW | NATA |  | － | NVTS | － | NDTS | NSSN | NITV |  | NVTN | NCTE | NISI | NKTL | NRTG | NATY |
| PEDV/XinJiang/2 | NSTW | NATA |  | － | NVTS | NKTI | NDTS | NSSN | NITV |  | NVTN | NCTE | NISI | NKTL | NRTG | NATY |
| PEDV/YunNan | NSTW | NATA |  | － | NVTS | － | NDTS | NSSN | NITV |  | NVTN | NCTE | NISI | NKTL | NRTG | NATY |

**Supplementary Table 5. Prediction results of potential specific *N*-glycosylation sites in the S protein of 15 PEDV strains compared with the vaccine strain.**

**Supplementary Fig 1**

**
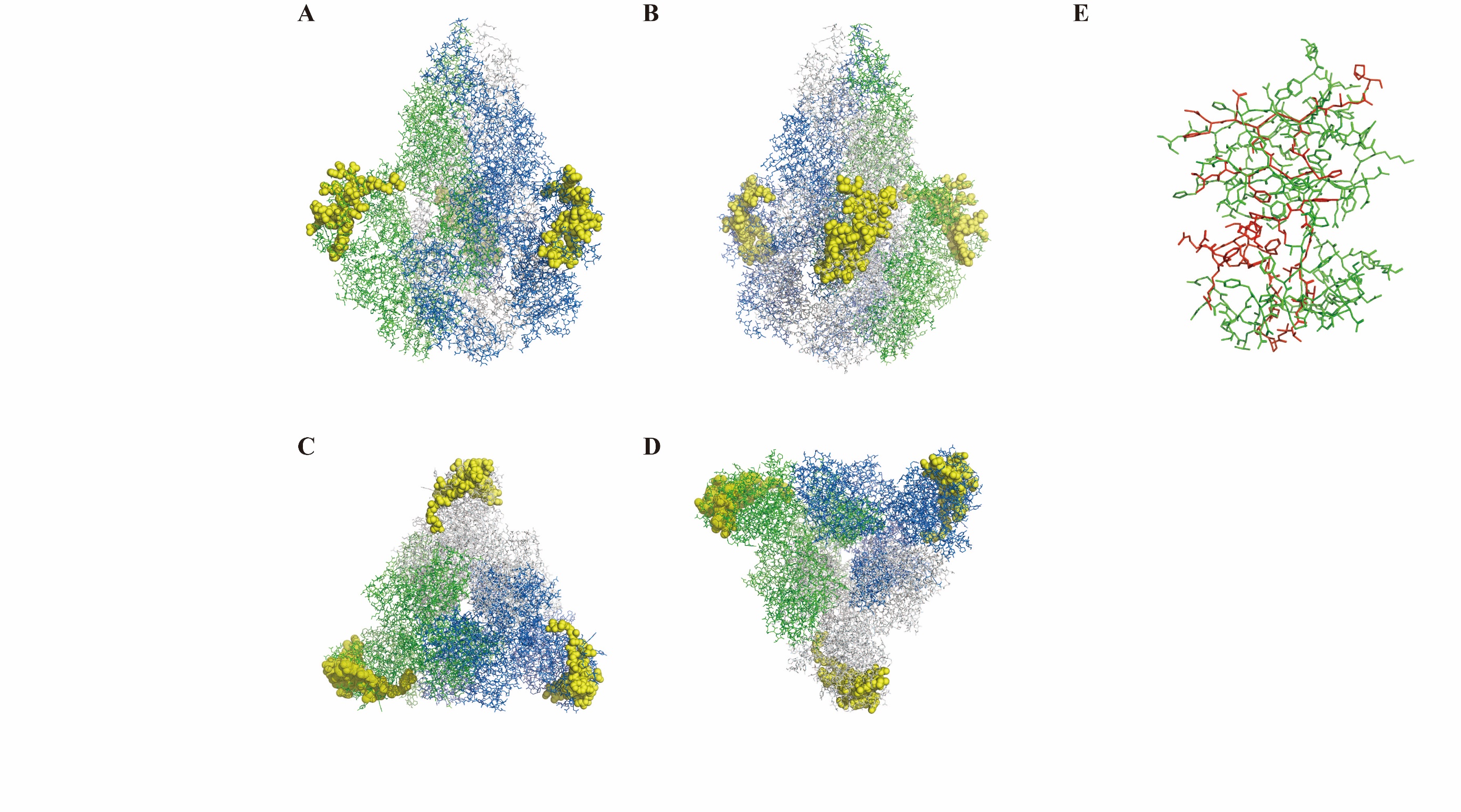
**

**Supplementary Fig 2**


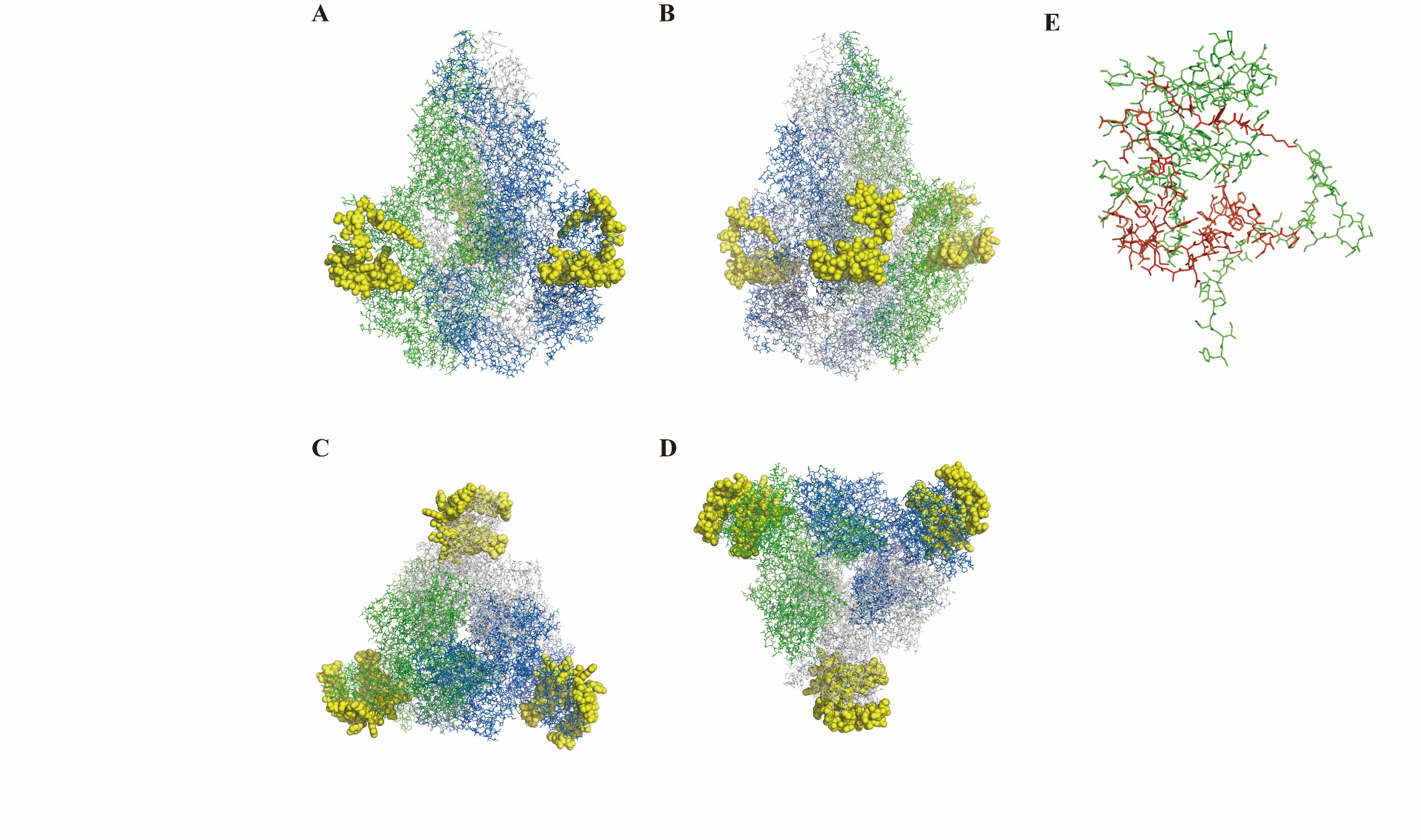


**Supplementary Fig 3**

**
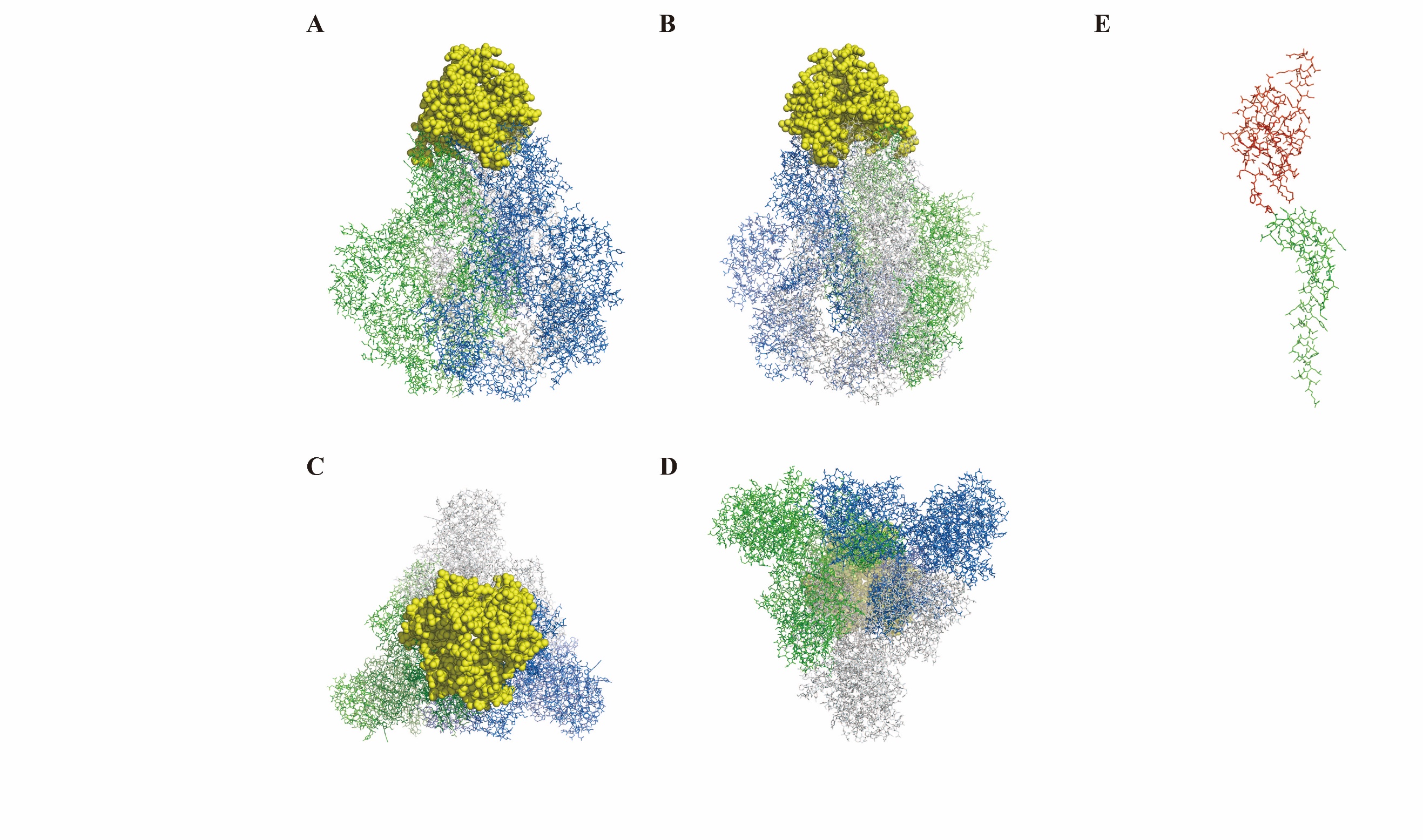
**

**Supplementary Fig 4**

**
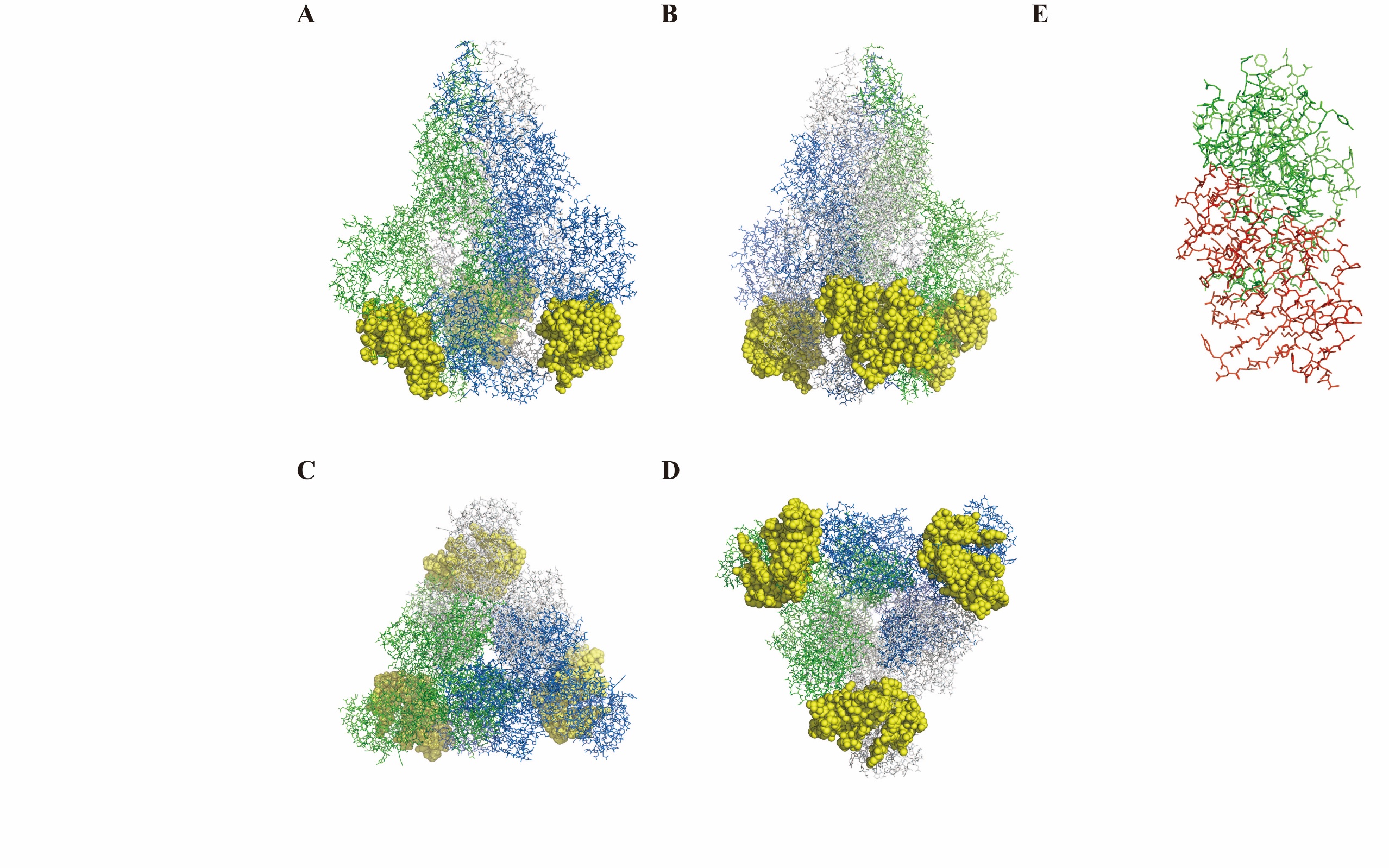
**

**Supplementary Fig 5**

**
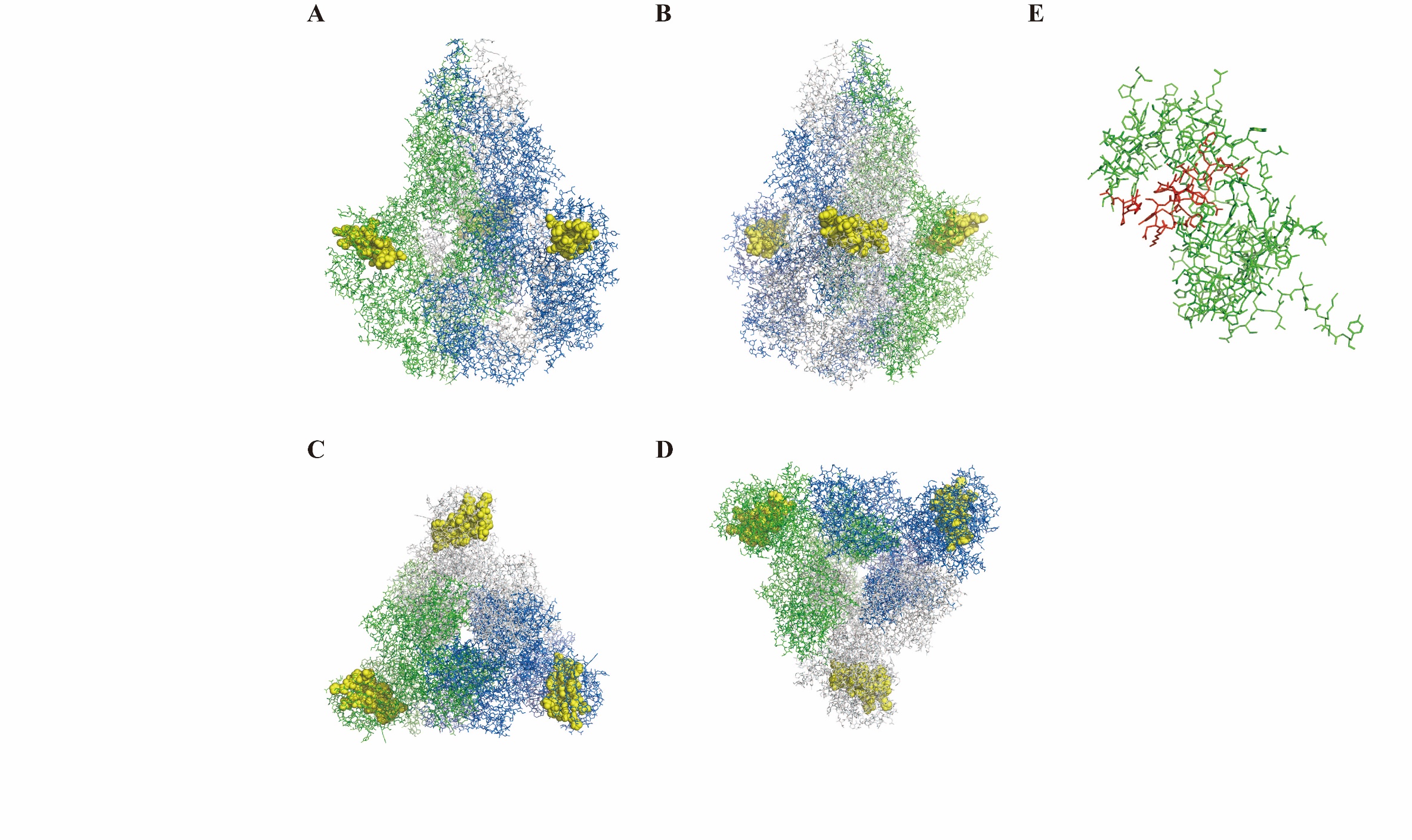
**

**Supplementary Fig 6**

**
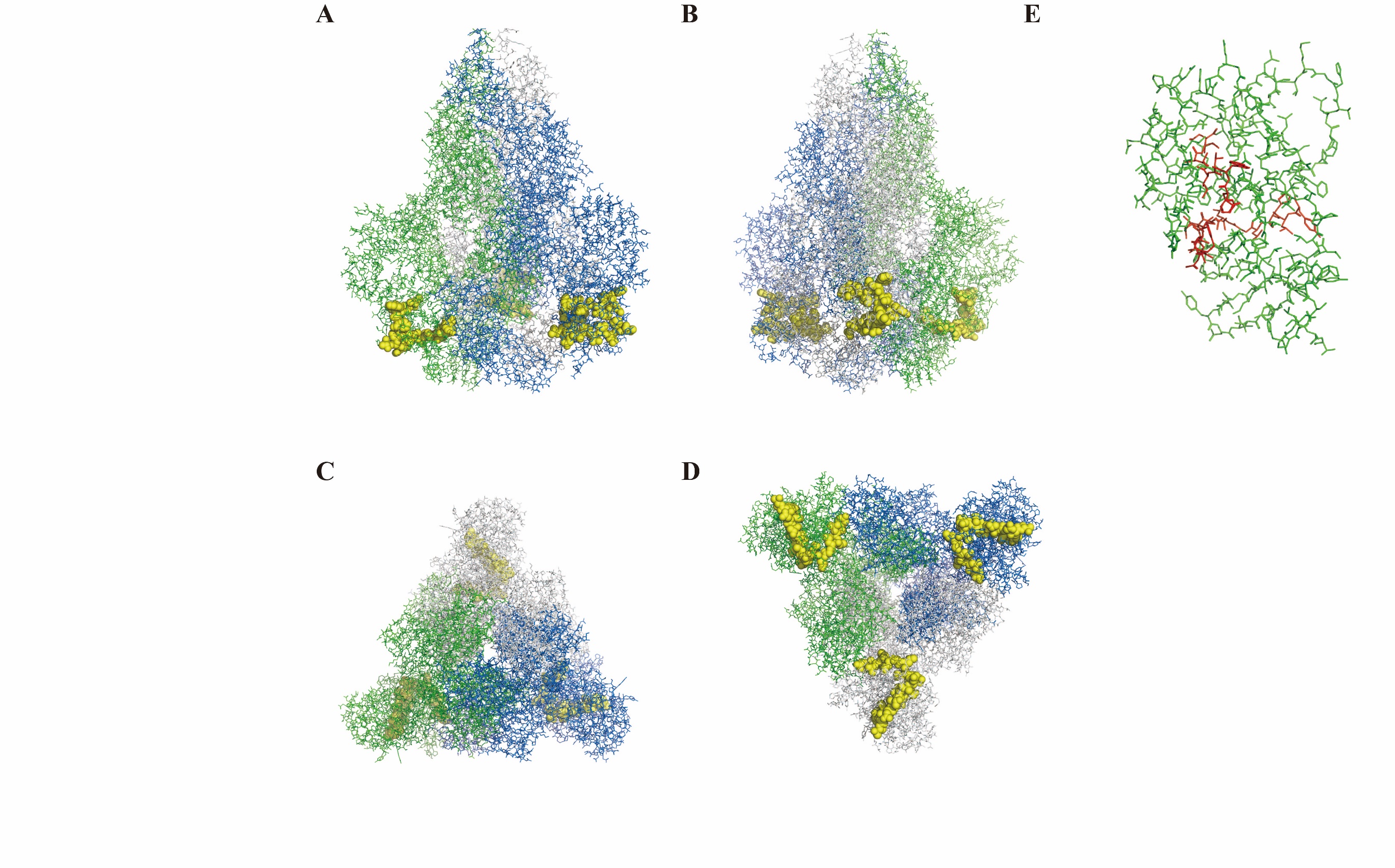
**

**Supplementary Fig 7**

**
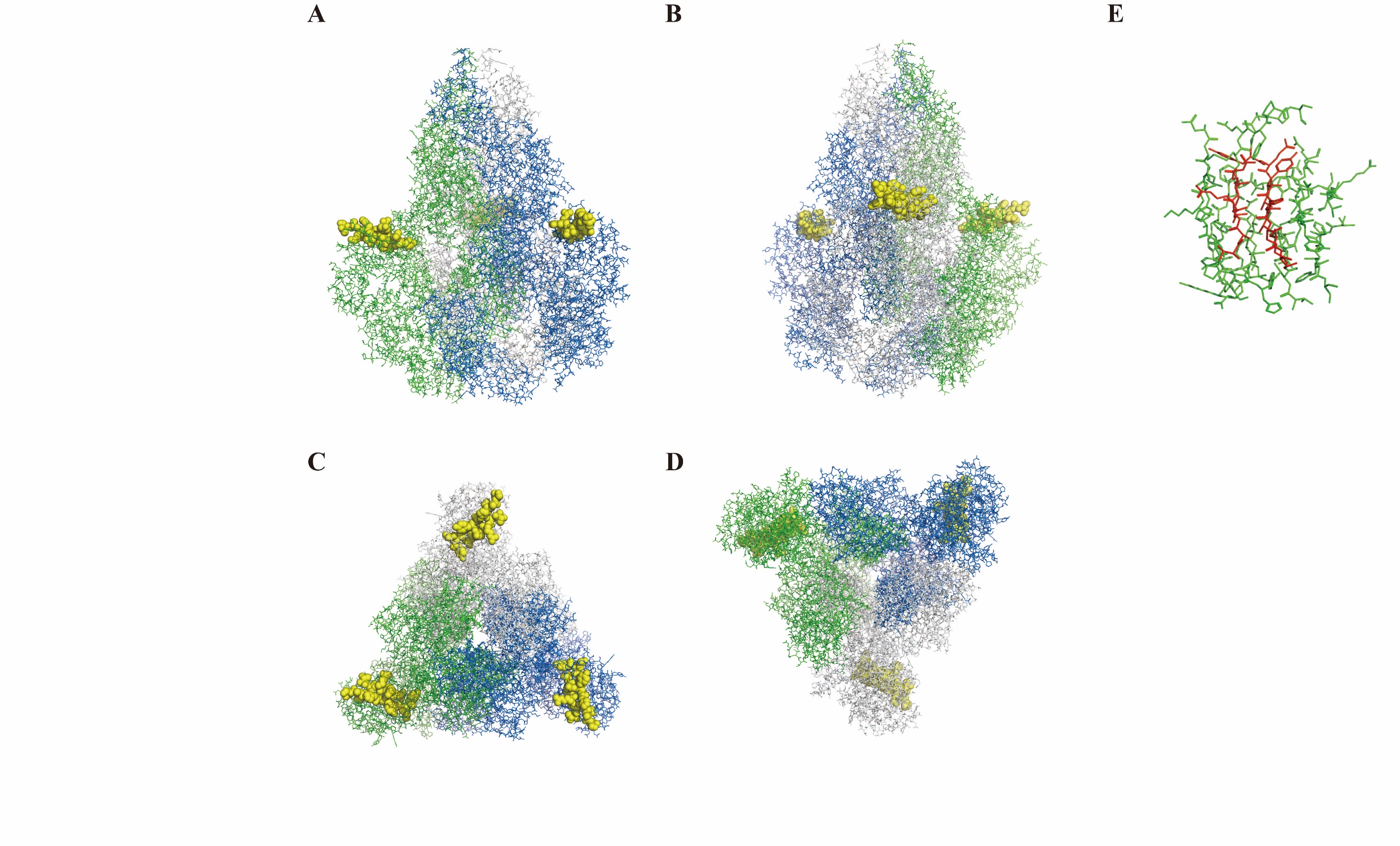
**

**Supplementary Fig 8**

**
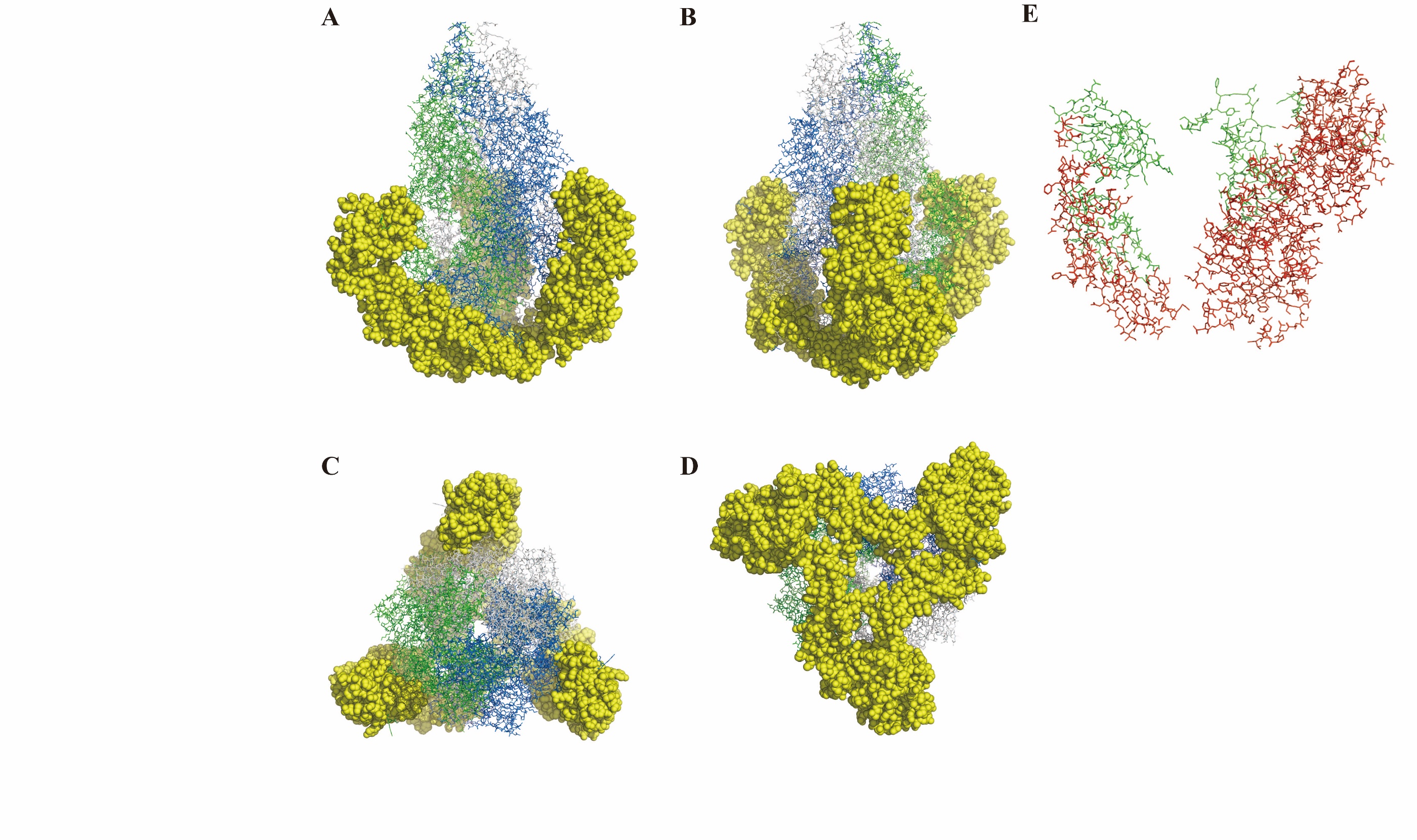
**

**Supplementary Fig 9**

**
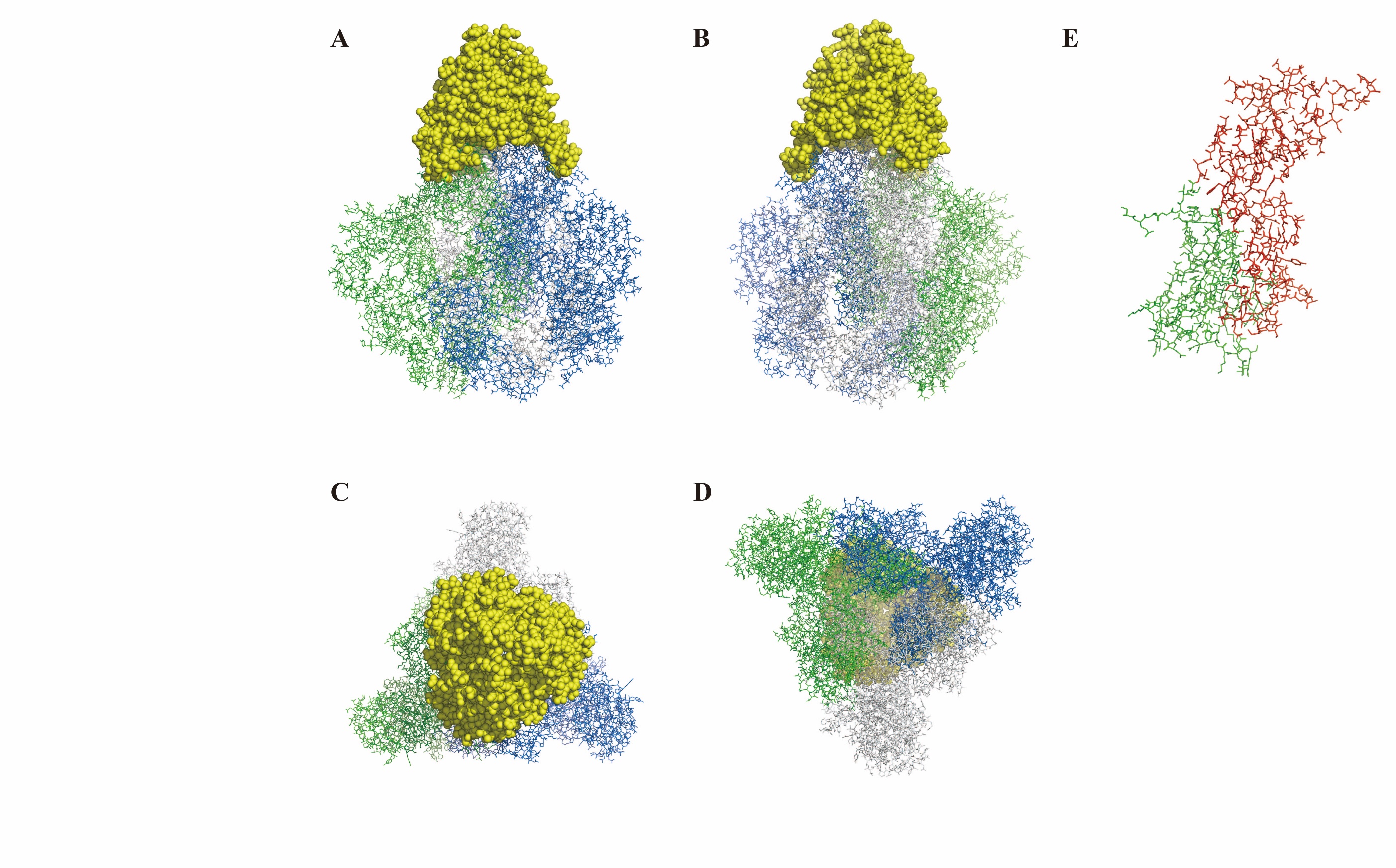
**
